# Peri-tumoural spatial distribution of lipid composition and tubule formation in breast cancer

**DOI:** 10.1186/s12885-022-09362-1

**Published:** 2022-03-17

**Authors:** Kwok-Shing Chan, Sai Man Cheung, Nicholas Senn, Ehab Husain, Yazan Masannat, Steven Heys, Jiabao He

**Affiliations:** 1 Institute of Medical Sciences, School of Medicine, University of Aberdeen, Aberdeen, UK; 2 Donders Institute for Brain, Cognition and Behaviour, Radboud University, Nijmegen, Netherlands; 3 Pathology Department, Aberdeen Royal Infirmary, Aberdeen, UK; 4 Breast Unit, Aberdeen Royal Infirmary, Aberdeen, UK

**Keywords:** Heterogeneity, Skewness, Entropy, Kurtosis, Monounsaturated fatty acids (MUFA)

## Abstract

**Background:**

Response guided treatment in breast cancer is highly desirable, but the effectiveness is only established based on residual cellularity from histopathological analysis after surgery. Tubule formation, a key component of grading score, is directly associated with cellularity, with significant implications on prognosis. Peri-tumoural lipid composition, a potential marker, can be rapidly mapped across the entire breast using novel method of chemical shift-encoded imaging, enabling the quantification of spatial distribution. We hypothesise that peri-tumoural spatial distribution of lipid composition is sensitive to tumour cellular differentiation and proliferative activity.

**Methods:**

Twenty whole tumour specimens freshly excised from patients with invasive ductal carcinoma (9 Score 2 and 11 Score 3 in tubule formation) were scanned on a 3 T clinical scanner (Achieva TX, Philips Healthcare). Quantitative lipid composition maps were acquired for polyunsaturated, monounsaturated, and saturated fatty acids (PUFA, MUFA, SFA). The peri-tumoural spatial distribution (mean, skewness, entropy and kurtosis) of each lipid constituent were then computed. The proliferative activity marker Ki-67 and tumour-infiltrating lymphocytes (TILs) were assessed histologically.

**Results:**

For MUFA, there were significant differences between groups in mean (*p* = 0.0119), skewness (*p* = 0.0116), entropy (*p* = 0.0223), kurtosis (*p* = 0.0381), and correlations against Ki-67 in mean (*ρ* = -0.5414), skewness (*ρ* = 0.6045) and entropy (*ρ* = 0.6677), and TILs in mean (*ρ* = -0.4621). For SFA, there were significant differences between groups in mean (*p* = 0.0329) and skewness (*p* = 0.0111), and correlation against Ki-67 in mean (*ρ* = 0.5910). For PUFA, there was no significant difference in mean, skewness, entropy or kurtosis between the groups.

**Conclusions:**

There was an association between peri-tumoural spatial distribution of lipid composition with tumour cellular differentiation and proliferation. Peri-tumoural lipid composition imaging might have potential in non-invasive quantitative assessment of patients with breast cancer for treatment planning and monitoring.

**Supplementary Information:**

The online version contains supplementary material available at 10.1186/s12885-022-09362-1.

## Background

Breast cancer is a major and expanding health challenge [1] with current incident rate at 15% and projected to reach 17% by 2035 [2]. Neoadjuvant chemotherapy is increasingly applied to improve treatment outcome [3] with effectiveness determined after surgery based on residual cellularity [4], and hence imaging markers sensitive to cellularity is central to response guided treatment. Tubule formation, an indicator of glandular differentiation and cellularity, is a marker for the degree of loss of well-defined tubular structures with open central lumina [5]. Tubule formation, a component in grade scores together with nuclear and mitotic count, is associated with elevated growth of capillary endothelial cells [6], the promotion of angiogenesis [7], poorer recurrence free survival [8] and cancer specific survival [9] in breast cancer. Tumour increases catabolism of tumour-surrounding adipocytes, leading to white and brown adipose tissue differentiation [10]. The elevated fatty acids metabolites are associated with prognostic features of grading scores [11]. Accurate tubular score, relying on the entire tumour rather than the periphery for nuclear and mitotic scores, imposes strict morphological criteria in breast tumour grading [5]. Hence, peri-tumoural lipid composition might be an imaging target as a measure of tubule formation to facilitate response guided treatment.

Lipid composition quantification using biochemical method of solvent extraction suffers from complex procedures, invasive nature and single spatial location [12]. Non-invasive lipid composition quantification method of correlation spectroscopy (COSY) [13] is limited to a single spatial location (single voxel) with a lengthy acquisition for a 2D spectral map [14]. Lipid composition mapping method of chemical shift imaging suffers from long acquisition time, low spatial resolution and subsequent undesirable transfer of signals between adjacent voxels, hampering clinical adoption [15]. Novel method of chemical shift-encoded imaging, an extension of conventional Dixon method for rapid overall lipid mapping [16], harnesses the known signal characteristics of the triglycerides model to quantitatively map lipid constituents [17, 18]. Chemical shift-encoded imaging enables the rapid mapping of lipid composition with adequate accuracy in adipose tissue [17, 18] and liver [19], making it feasible to examine the role of peri-tumoural lipid composition in breast cancer [20, 21] within a cohort size targeting at personalised care.

We therefore hypothesise that the peri-tumoural spatial distribution of lipid composition from chemical shift-encoded imaging is sensitive to tubule formation, and is associated with proliferative activities.

## Methods

**Fig. 1:**
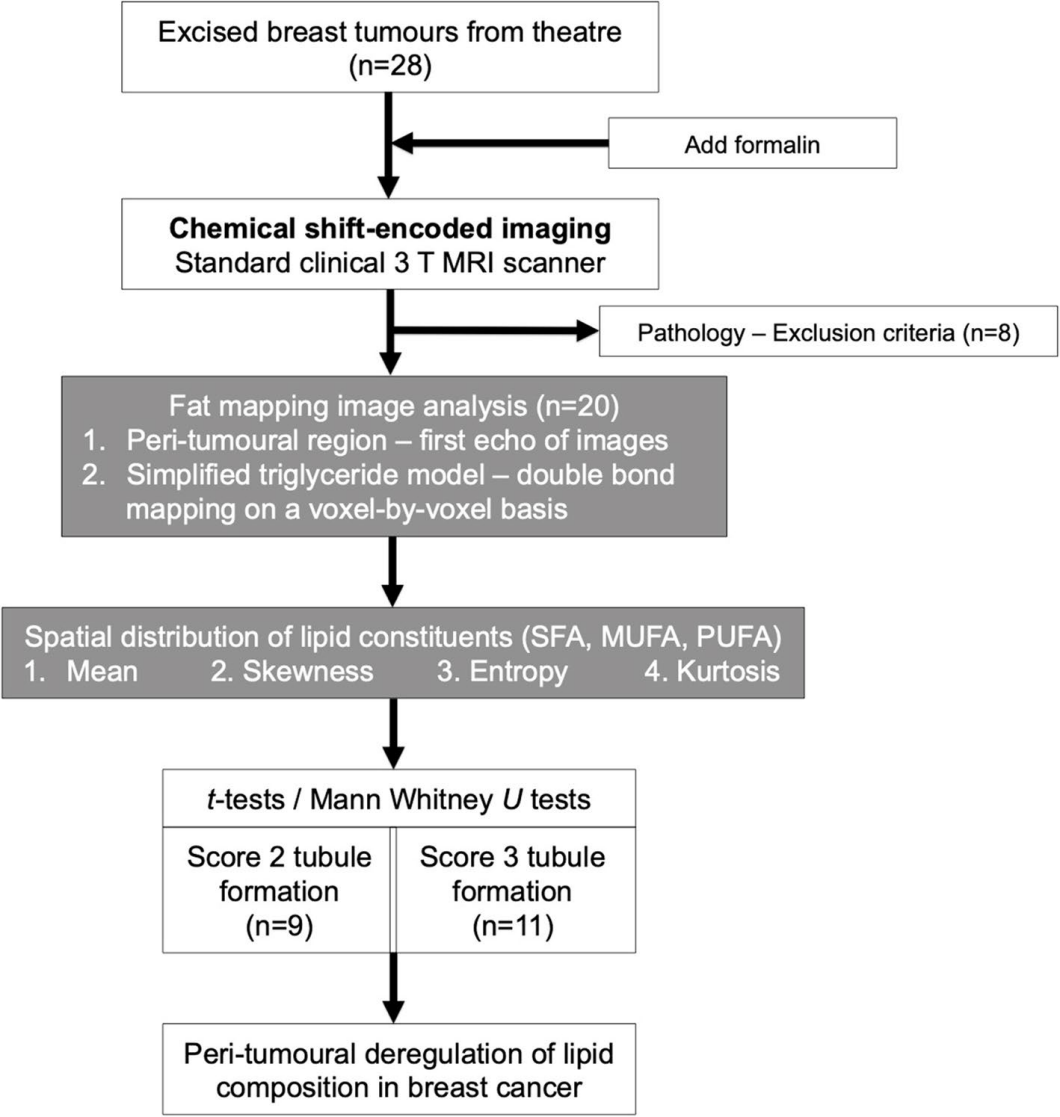
Twenty specimens (9/11 for tubule formation Scores 2/3) freshly excised from patients with invasive carcinoma were examined, with proliferative activity marker Ki-67, tumour-infiltrating lymphocytes (TILs) and Nottingham Prognostic Index assessed histologically. Lipid composition maps were acquired using chemical shift-encoded imaging on standard clinical 3 T MRI scanner. The peri-tumoural region was delineated on the first echo of lipid composition images, and adipose voxels within the region were extracted from lipid composition maps to quantify mean, skewness, entropy and kurtosis. Independent sample *t*-tests and Mann Whitney *U* tests were conducted between groups, and Spearman’s rank correlation tests performed against prognostic markers

To probe this hypothesis, we conducted a two-group cross-sectional study examining the peri-tumoural spatial distribution of lipid composition in whole tumour specimens freshly excised from patients (Fig. 1). The study was approved by the North West – Greater Manchester East Research Ethics Committee (REC Reference: 16/NW/0221), and written informed consents were obtained from all the participants prior to the study.

### Clinical procedure

Twenty patients (mean age 57 years, range 35 – 78 years, 9 Score 2 and 11 Score 3 in tubule formation) with invasive breast carcinoma participated in the study. Patients undergoing breast conservation surgery, with tumour size larger than 10 mm in diameter on mammography, with no previous malignancies, chemotherapy or radiotherapy prior to surgery were eligible. For accurate delineation of peri-tumoural adipose tissue, a tumour size larger than 10 mm was required to ensure adequate image resolution. The majority of breast tumours that are larger than 10 mm would have tubule formation Score 2 and 3. To avoid highly skewed patient distribution leading to biased results, tubule formation score 1 was hence excluded from the study. The study was completed between September 2016 and February 2018.

The specimen, upon excision, was submerged in 10% formalin to prevent tissue degradation and immobilised using a custom-built holding harness in a sealed container. The tissue specimen was immediately transported from the operating theatre to Aberdeen Biomedical Imaging Centre for lipid composition mapping. Routine clinical histopathological examination was subsequently performed to determine histological tumour size, grade and Nottingham Prognostic Index (NPI) [22]. Tumour cellular proliferation was assessed quantitatively using proliferative activity marker Ki-67 [23], and protumourigenic property semiquantitatively using tumour-infiltrating lymphocytes (TILs) [24] by a consultant pathologist (EH) after single batch immunostaining [22].

### Lipid composition mapping

Images were acquired on a 3 T whole-body clinical MRI scanner (Achieva TX, Philips Healthcare, Best, Netherlands), using a 32-channel receiver coil for signal detection and a body coil for uniform transmission. Lipid composition images were acquired using chemical shift-encoded imaging sequence [17, 18] with an isotropic resolution of 2.2 mm, initial TE of 1.14 ms, echo spacing of 1.14 ms, 16 echoes, TR of 20 ms, flip angle of 6° and 9 signal averages.

Image analysis was conducted in MATLAB (MathWorks Inc., Natick, MA, USA). The maps of the number of double bonds in triglycerides were computed from raw data [17], before subsequent calculation of quantitative maps of polyunsaturated fatty acids (PUFA), monounsaturated FA (MUFA) and saturated FA (SFA) as a percentage of the total amount of lipids [17, 18]. The peri-tumoural region was delineated on the first echo of lipid composition images, and adipose voxels (lipid signal over 60% of total signal) within the region were extracted from lipid composition maps for histogram analysis. The spatial distribution (mean, skewness, entropy and kurtosis [25, 26]) were subsequently computed based on the histogram distribution for each lipid constituent. The coefficient of variance (CoV) of lipid composition mapping was 3.5%, 3.4% and 2.2% for PUFA, MUFA and SFA respectively observed in a sunflower oil phantom. Full details of data acquisition, data processing, validation in oil phantoms and typical lipid constituent maps are given in Electronic Supplementary Material (see Additional file 1).

### Statistical analysis

All statistical analysis was performed in the SPSS software (Release 24.0, SPSS Inc., Chicago, IL, USA). Shapiro–Wilk test for normality was performed on all the collected data. Descriptive statistics were computed for peri-tumoural lipid composition in Luminal A, Luminal B [human epidermal growth factor receptor 2 (HER2) negative] (Luminal B-HER2(-)), Luminal B-HER2( +) and triple negative (TN) breast cancer. Group comparisons (independent sample *t*-tests and Mann Whitney *U* tests depending on the normality of sample distribution) were performed to compare the peri-tumoural spatial distribution of lipid constituents between tubule formation Scores. The Spearman’s rank correlation tests were performed between the spatial distribution in each lipid constituent against proliferative activity marker Ki-67 and TILs. A *p*-value < 0.05 was considered statistically significant.

## Results

**Fig. 2:**
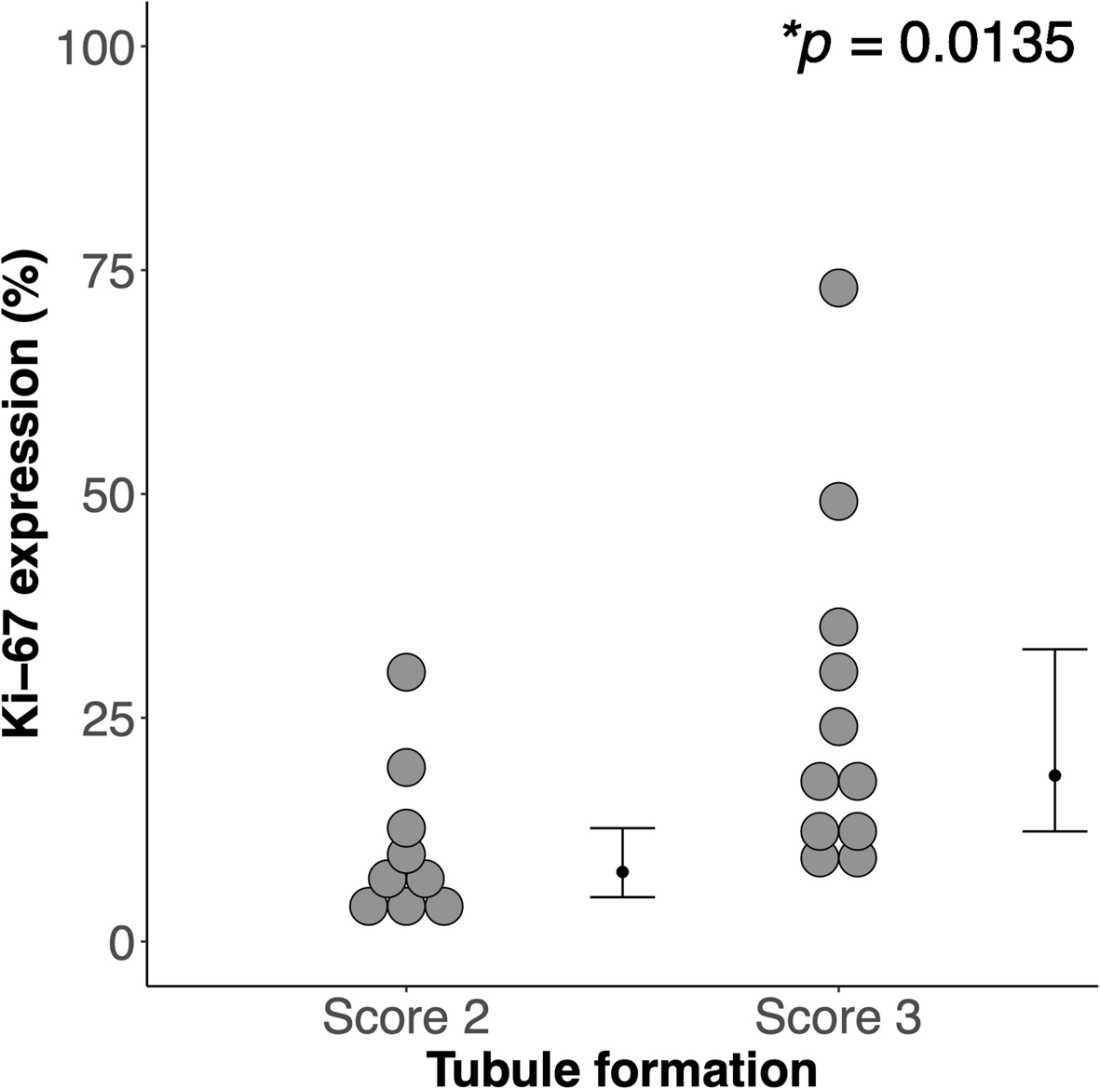
The group difference in tumour proliferative activity marker Ki-67 between tubule formation Score 2 and Score 3 breast cancer (*n* = 9,11). Each dot represents the expression of Ki-67 (in percentage), and the dots are organised in two columns corresponding to Scores 2 and 3. The distributions were not normally distributed and the error bars indicate median (interquartile range). The 2-tailed Mann Whitney *U* test was performed between the groups and *p*-value is shown. Statistically significant *p* value (< 0.05) is marked by ‘*’

**Fig. 3:**
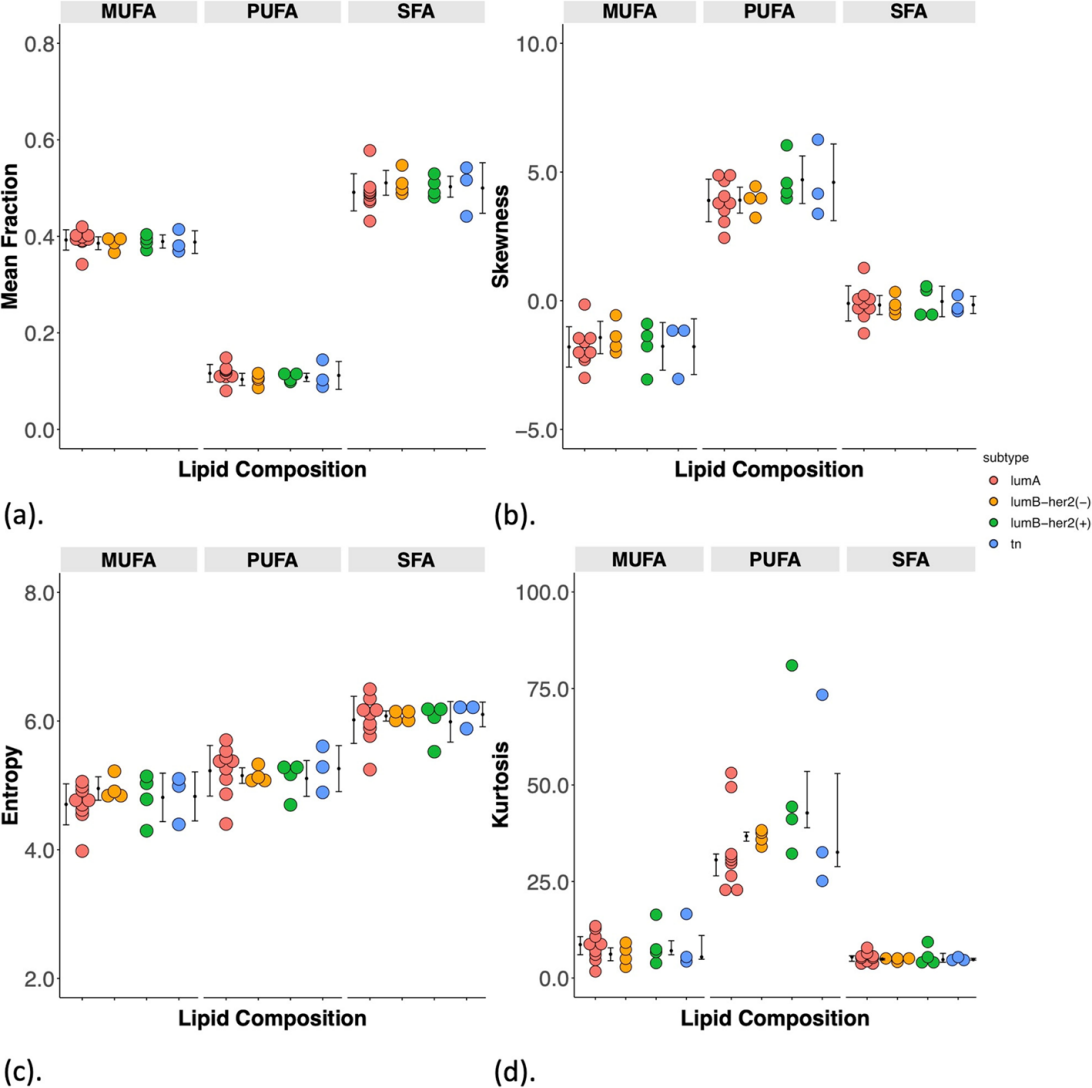
The group difference in (**a**) Mean, (**b**) Skewness, (**c**) Entropy and (**d**) Kurtosis of MUFA, PUFA, SFA among Lumina A (*n* = 9), Luminal B-HER2(-) (*n* = 4), Luminal B-HER2( +) (*n* = 4) and triple negative (TN) breast cancer (*n *= 3). Each dot represents a peri-tumoural mean fraction or spatial distribution, and the dots are organised in four columns corresponding to molecular subtypes. All metrics, apart from kurtosis were normally distributed and the error bars indicate the mean ± SD (median (interquartile range) for kurtosis)

Breast tumours with tubule formation Score 3 have significantly higher tumour proliferative activity (Table 1, Fig. 2). There was no significant difference in age and tumour size between tubule formation Scores. The peri-tumoural lipid composition in Luminal A, Luminal B-HER(-), Luminal B-HER( +) and TN breast cancer are shown in Fig. 3 and Table 2.

**Table 1:** Descriptive statistics of breast cancer patients with tubule formation Score 2 and 3 are shown for each group and the entire cohort. Values are expressed as mean and standard deviation, apart from Nottingham Prognostic Index (NPI) and proliferative activity marker Ki-67 reported as median and interquartile range. Pathological entries are expressed as number of positive observations

Demographic	All (*n* = 20)	Tubule formation		*P*-value
		**Score 2 (*n* = 9)**	**Score 3 (*n* = 11)**	
Age (years)	57 ± 14	62 ± 13	54 ± 14	0.222
Tumour Size (mm)	26 ± 5	27 ± 7	26 ± 5	0.518
Nottingham Prognostic Index (NPI)	4.44 (3.5 – 4.59)	3.54 (3.5 – 4.48)	4.48 (4.42 – 5.16)	0.094
Histological grade				
II	10	7	3	0.070
III	10	2	8	
Lymph node involvement	4	1	3	0.591
Ki-67	12.85 (8.31 – 25.54)	7.78 (4.96 – 12.68)	18.56 (12.32 – 32.65)	0.014*
Molecular subtypes				
Luminal A	9	6	3	0.175
Luminal B-HER2 negative	4	2	2	1.000
Luminal B-HER2 positive	4	1	3	0.591
Triple Negative	3	0	3	0.218

*HER* 2 Human epidermal growth factor receptor 2

**Table 2:** Peri-tumoural monounsaturated, polyunsaturated and saturated fatty acids (MUFA, PUFA, SFA) mean, skewness, entropy and kurtosis were compared between molecular subtypes and in tubule formation Scores 2 and 3. The Spearman’s rank correlation coefficients (*ρ*) between lipid constituents against proliferative activity marker Ki-67 and tumour-infiltrating lymphocytes (TILs) are also shown. Statistical significant differences (*p* < 0.05) are marked by ‘*’

**Lipid**	**Spatial distribution measures**	**Molecular subtypes**				**Tubule formation**		***P***	**Ki-67**		**TILs**	
		**Luminal A (*n* = 9)**	**Luminal B-HER2(-) (*n* = 4)**	**Luminal B-HER( +) (*n* = 4)**	**Triple Negative (*n* = 3)**	**Score 2 (*n* = 9)**	**Score 3 (*n* = 11)**		*ρ*	***p***	*ρ*	***p***
***MUFA***	Mean	0.39 ± 0.02	0.39 ± 0.01	0.39 ± 0.01	0.39 ± 0.02	0.40 ± 0.01	0.38 ± 0.02	0.012*	-0.54	0.014*	-0.46	0.040*
	Skewness	-1.80 ± 0.78	-1.43 ± 0.63	-1.77 ± 0.93	-1.79 ± 1.08	-2.17 ± 0.55	-1.35 ± 0.75	0.012*	0.60	0.005*	0.42	0.066
	Entropy	4.71 ± 0.32	5.00 ± 0.18	4.81 ± 0.38	4.83 ± 0.38	4.62 ± 0.30	4.94 ± 0.24	0.022*	0.67	0.001*	0.32	0.169
	Kurtosis	8.64 (6.04 – 10.71)	6.18 (4.46 – 7.82)	7.08 (6.02 – 9.67)	5.44 (4.86 – 11.01)	8.91 (7.37 – 12.80)	5.44 (4.08 – 7.21)	0.038*	-0.44	0.054	-0.40	0.080
***PUFA***	Mean	0.12 ± 0.02	0.10 ± 0.01	0.11 ± 0.01	0.11 ± 0.03	0.12 ± 0.01	0.11 ± 0.02	0.099	-0.56	0.010*	-0.33	0.161
	Skewness	3.90 ± 0.83	3.91 ± 0.50	4.70 ± 0.92	4.60 ± 1.49	4.12 ± 1.04	4.20 ± 0.85	0.852	0.10	0.682	0.02	0.923
	Entropy	5.23 ± 0.39	5.15 ± 0.12	5.11 ± 0.28	5.26 ± 0.36	5.16 ± 0.25	5.22 ± 0.36	0.708	-0.15	0.540	-0.06	0.803
	Kurtosis	30.60 (26.46 – 32.11)	36.77 (35.44 – 37.79)	42.76 (38.92 – 53.52)	32.58 (28.86 – 52.97)	38.29 (31.42 – 49.45)	32.58 (27.44 – 36.77)	0.295	0.28	0.230	-0.20	0.404
***SFA***	Mean	0.49 ± 0.04	0.51 ± 0.03	0.50 ± 0.02	0.50 ± 0.05	0.48 ± 0.02	0.51 ± 0.04	0.033*	0.59	0.006*	0.37	0.107
	Skewness	-0.11 ± 0.68	-0.17 ± 0.37	-0.03 ± 0.59	-0.16 ± 0.33	0.22 ± 0.53	-0.39 ± 0.37	0.011*	-0.42	0.067	-0.26	0.259
	Entropy	6.02 ± 0.37	6.08 ± 0.08	5.99 ± 0.32	6.10 ± 0.19	5.96 ± 0.21	6.10 ± 0.32	0.272	0.18	0.450	0.08	0.738
	Kurtosis	5.45 (4.34 – 5.75)	4.90 (4.68 – 5.04)	4.76 (4.08 – 6.39)	4.78 (4.65 – 5.10)	5.45 (4.95 – 6.43)	4.78 (4.09 – 5.36)	0.067	-0.45	0.049*	-0.32	0.165

**Fig. 4:**
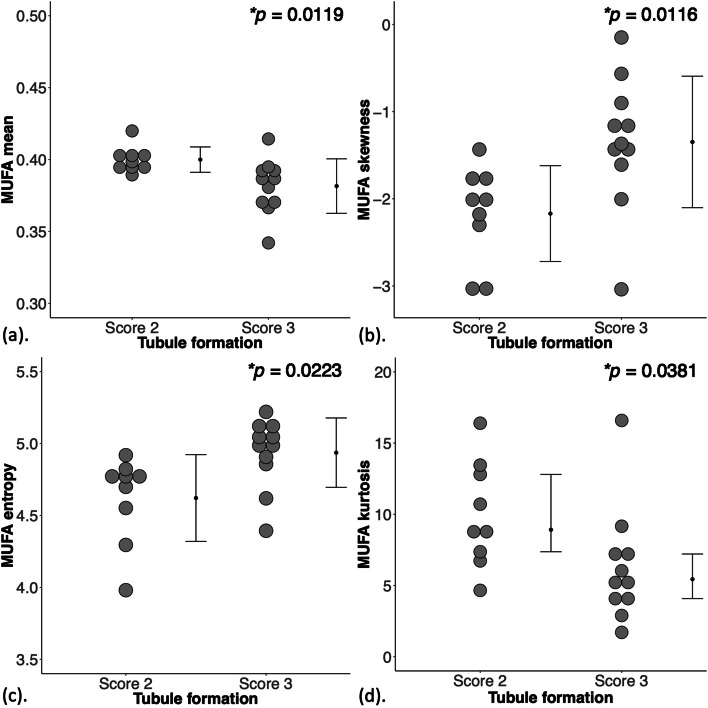
The group difference in (**a**) Mean (*n* = 9,11), (**b**) Skewness (*n* = 9,11), (**c**) Entropy (*n* = 9,11) and (**d**) Kurtosis *(n *= 9,11) of MUFA are shown in dot plots. Each dot represents a peri-tumoural spatial distribution, and the dots are organised in two columns corresponding to tubule formation Scores. All distributions, apart from kurtosis were normally distributed and the error bars indicate the mean ± SD (median (interquartile range) for kurtosis). The 2-tailed independent sample *t*-tests were performed between the groups and *p*-value is shown for each plot. Statistically significant *p* values (< 0.05) are marked by ‘*’

**Fig. 5:**
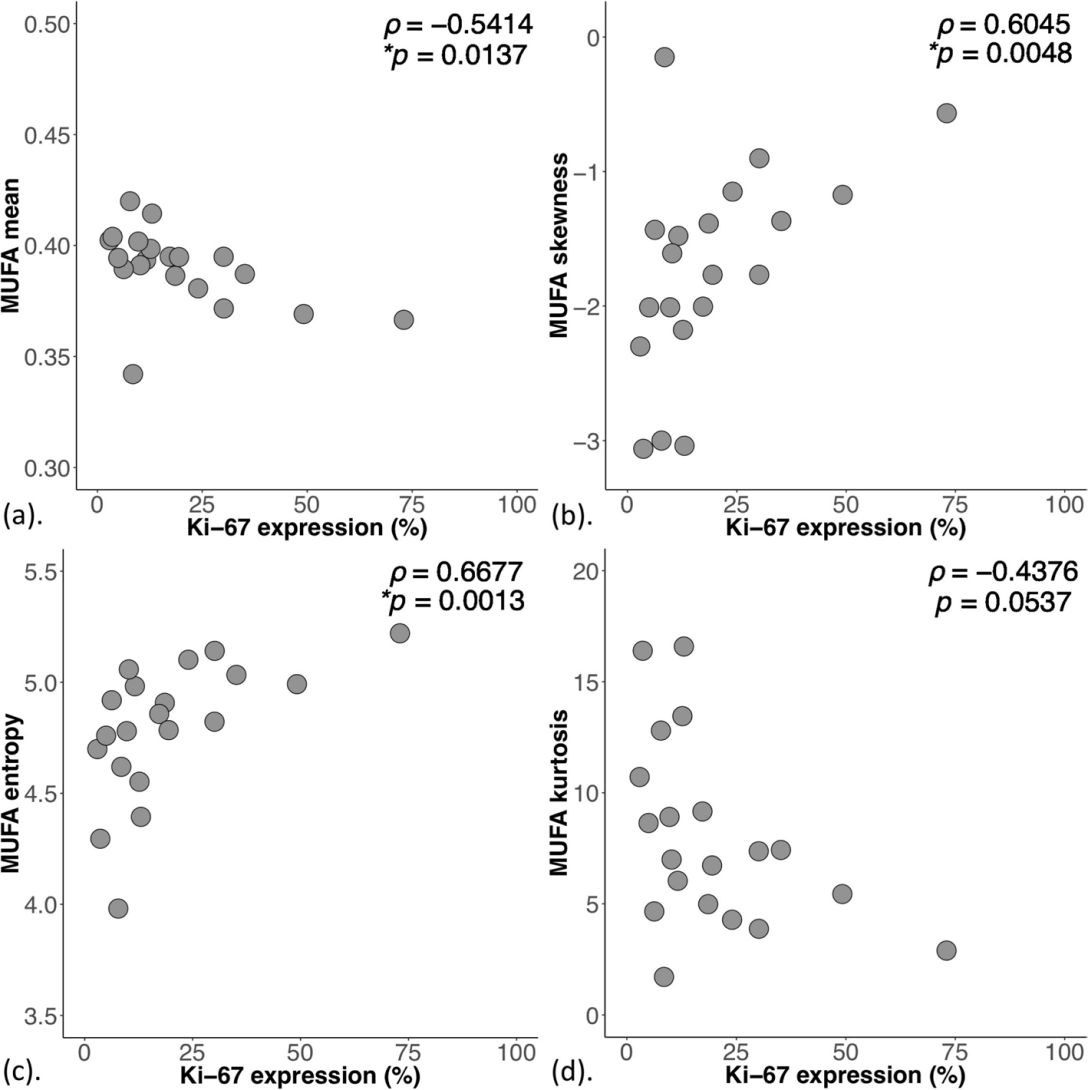
The correlation of (**a**) Mean (*n* = 20), (**b**) Skewness (*n* = 20), (**c**) Entropy *(n* = 20) and (**d**) Kurtosis (*n* = 20) of MUFA against proliferative activity marker Ki-67 are shown in scatter plots. Spearman’s rank correlation (*rho*), appropriate for non-linear monotonic relationship, was used for correlation analysis and respective *ρ* score and *p*-value is shown for each plot. Statistically significant *p* values (< 0.05) are marked by ‘*’

For MUFA, there was a significantly lower mean (0.38 ± 0.02, *p* = 0.012, Fig. 4a, Table 2), higher skewness (negative, -1.35 ± 0.75, *p* = 0.012, Fig. 4b), higher entropy (4.94 ± 0.24, *p* = 0.022, Fig. 4c), and a significantly lower kurtosis (5.44 (4.08 – 7.21), *p* = 0.038, Fig. 4d) in Score 3. For MUFA against Ki-67, there were significant correlations (Spearman’s rank, *ρ*) in mean (*ρ* = -0.54, *p* = 0.014, Fig. 5a, Table 2), skewness (*ρ* = 0.60, *p* = 0.005, Fig. 5b), entropy (*ρ* = 0.67, *p* = 0.001, Fig. 5c), but not in kurtosis (Fig. 5d). For MUFA against TILs, there was significant correlation (Spearman’s rank) in mean (*ρ* = -0.46, *p* = 0.040, Table 2).

**Fig. 6:**
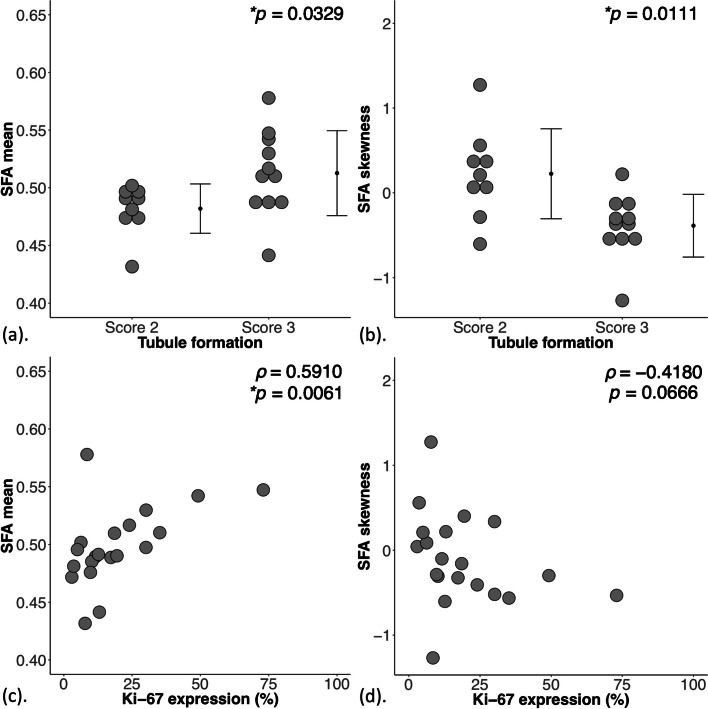
The group difference in (**a**) Mean *(n *= 9,11) and (**b**) Skewness (*n* = 9,11) of SFA are shown in dot plots. Each dot represents a peri-tumoural spatial distribution, and the dots are organised in two columns corresponding to tubule formation Scores. All distributions shown were normally distributed and the error bars indicate the mean and standard deviation. The 2-tailed independent sample *t*-tests were performed between the groups and *p* value is shown for each plot. The Spearman’s rank correlation (*rho*) of (**c**) Mean (*n* = 20) and (**d**) Skewness (*n* = 20) of SFA against proliferative activity marker Ki-67 are shown in scatter plots. Statistically significant *p* values (< 0.05) are marked by ‘*’

For SFA, there was a significantly higher mean (0.51 ± 0.04, *p* = 0.033, Fig. 6a, Table 2) and lower skewness (negative, -0.39 ± 0.37, *p* = 0.011, Fig. 6b) in Score 3, but not in entropy or kurtosis. For SFA against Ki-67, there was significant correlation (Spearman’s rank) in mean (*ρ* = 0.59, *p* = 0.006, Fig. 6c, Table 2), but not in skewness (Fig. 6d). For SFA against TILs, there were no significant correlations in mean or skewness.

For PUFA, there were no significant differences in mean, skewness, entropy or kurtosis between the groups (Table 2).

**Fig. 7:**
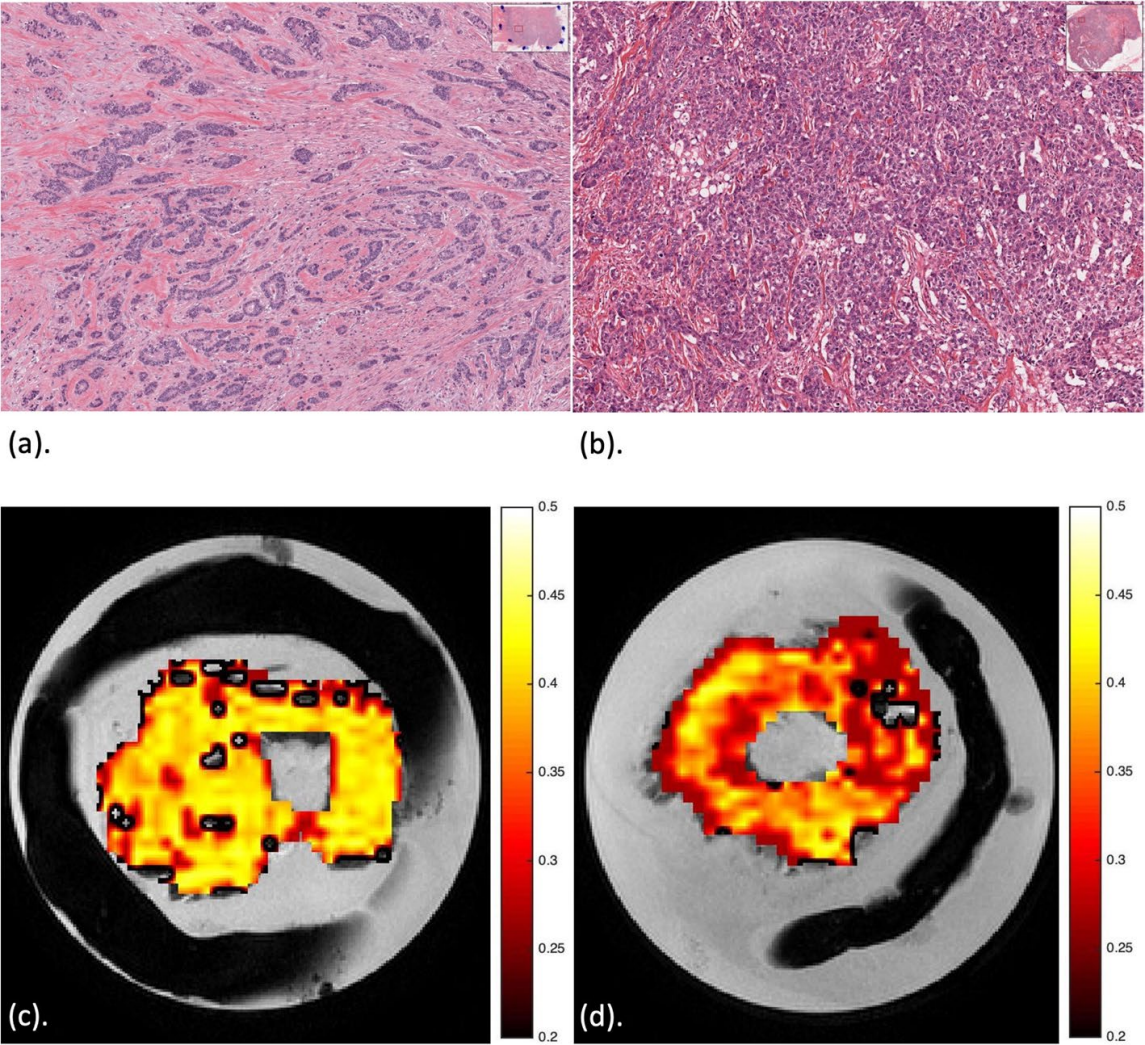
Haematoxylin and eosin (H & E) stained microscopy slides from breast cancer specimens with the corresponding peri-tumoural MUFA imaging maps (overlaid on anatomical image). (**a**), (**c**) Tubule formation Score 2 and (**b**), (**d**) Tubule formation Score 3. A tubule formation Score 2 indicates 10 – 75% of the tumour has glandular differentiation in a tubular pattern, while tubule formation Score 3 indicates < 10% has tubular differentiation. Sections at the greatest dimension of typical specimen are shown. Magnification, × 10

Haematoxylin and eosin (H & E) stained microscopy slides from two typical invasive breast cancer specimens, one from tubule formation Score 2 and one from Score 3, are shown in Fig. 7 (a, b). The corresponding peri-tumoural MUFA maps are shown in Fig. 7 (c, d).

## Discussion

In this work, we found significant differences in all the spatial distribution measures of peri-tumoural MUFA between tubule formation Scores, with strong association with proliferative activity marker Ki-67. There were significant differences in the measures of mean and skewness for SFA, with mean SFA associated with proliferative activity. There were no significant differences in the spatial distribution for PUFA between groups.

MUFA, supported by significant differences in all the measures of peri-tumoural spatial distribution, might hold the central role in tubule formation, with strong correlations against proliferative activity marker. For least glandular differentiation, a reduction in mean MUFA indicates lower overall peri-tumoural MUFA, potentially due to accelerated absorption of MUFA into tumour core to support aggressive cancer cell growth [27]. The negative skewness indicates an increase in peri-tumoural adipocytes with a reduction in MUFA, while the lower magnitude indicates a more homogeneous distribution of MUFA. Hence least glandular differentiation might be associated with a wider spread of adipocytes with a reduction in MUFA. The reduction in kurtosis, in consistency with skewness, indicates a more homogeneous spread of MUFA for least glandular differentiation, and represents a potential increase in MUFA transportation between peri-tumoural adipocytes and tumour [28]. Subsequently, there was an elevation of entropy due to the increase in the irregular and diverse clustering of MUFA in the peri-tumoural adipocytes [29]. The correlation in skewness and entropy of MUFA with Ki-67 showed consistent direction between group differences in tubule formation and Ki-67, with decreased glandular differentiation also reflecting a potential elevation of tumour proliferative activity and cellularity. Decreased glandular differentiation, associated with aggressive cancer cell growth, therefore might be associated with infiltration of peri-tumoural MUFA at the advancing edge [30], leading to a reduction in MUFA in the adipocytes [31]. Hence, the peri-tumoural spatial distribution of MUFA might be an imaging biomarker directly associated with tumour proliferation. Consequently, suppression of MUFA utilisation has been exploited by chemotherapeutic agents for promoting cancer apoptosis [32].

SFA, with limited number of significant differences between groups and correlations against proliferative activity marker, might hold secondary role in tubule formation, while PUFA showing no apparent connection. For least glandular differentiation, an elevation of mean SFA indicates higher overall peri-tumoural SFA, reflecting a potential extrusion of SFA from tumour to alleviate SFA-induced apoptosis (lipotoxicity) [31, 33] arising from de novo synthesis [28]. The transition from positive to negative skewness in SFA indicates a corresponding increase in adipocytes with higher SFA, reflecting a potential export of SFA into peri-tumoural space. The lack of significant differences in kurtosis and entropy of SFA might indicate a homogeneous export of SFA, with spatial distribution independent from tubule formation.

The correlation between mean SFA and proliferative activity marker Ki-67 might indicate a dependency of overall tumour growth on the export of SFA [28]. However, the lack of association in skewness of SFA against Ki-67 indicates tumour growth might not be dependent on the peri-tumoural spatial distribution. PUFA, although depleted in tumour core, might not be involved in the central lipid metabolism of de novo synthesis associated with tumour aggressiveness [27], leading to no apparent connection between peri-tumoural spatial distribution and tubule formation. The homogeneous elevation of peri-tumoural SFA could be positioned to facilitate early detection of breast cancer [28, 31], particularly valuable in screening high-risk BRCA1/2 genetic mutation carriers prone to deregulation in lipid saturation [13].

This work was the first investigation of the peri-tumoural spatial distribution of lipid composition in breast cancer, showing MUFA might be a central biomarker of glandular differentiation. Since lipid composition in breast is affected by oestrogen in premenopausal patients compared to postmenopausal [34], the association between peri-tumoural spatial distribution of lipid composition and glandular differentiation may hold regardless of the menopausal status. The ex vivo design prevented image corruption arising from motion-induced phase instability, while allowing the employment of high sensitivity hardware for enhanced resolution. Despite the submergence in formalin, the imaging was conducted on the same day of excision with negligible degradation of lipids (minimal even for three months) [35]. Benign nodules were not included due to insufficient surplus tissue samples from benign nodules. In the future, benign nodules such as adenosis should be collected to allow a direct comparison against breast tumour. Beyond the investigation of tubule formation, future work in intraductal epithelial abnormalities that might be precancerous and tumour initiation in high-risk population, will extend the clinical value of lipid composition mapping.

Lipid composition mapping, although limited by overlapping distribution between groups and low to moderate correlations with tumour proliferation, could be a valuable clinical research tool to support image guided treatment. Histopathological analysis to determine tubule formation is the clinical standard, however demands significant expertise. The association between tubule formation score and peri-tumoural lipid composition was based on group difference instead of correlation, and the lipid composition imaging proposed in this research is the first step towards in vivo patient application for treatment planning and monitoring. The imaging marker, although showing significant sensitivity to tubule formation, does not replace traditional histopathology using optical microscope as established clinical routine standard. However, the non-invasive nature of the imaging marker may contribute to improved patient care, particularly in the context of neoadjuvant setting for complementing clinical routine assessments. The association with Ki-67 is of considerable clinical relevance, however concordance with ER, PgR, HER2 positivity and histological grade remains critical for breast cancer treatment. The cohort size was small, limiting the reliability for evaluating the potential association of NPI and histological grade with molecular subtypes [36]. However, this proof of concept imaging study aimed at improving personalised care to derive critical tumour characteristics non-invasively before surgery based on established mechanistic understanding of breast cancer. Hence, the main thrust of this work was to identify sensitive imaging biomarkers in a relatively small patient cohort size. Future larger mechanistic studies are required to unravel the lipid regulation in breast cancer and allow multivariate analysis to probe the association with molecular and immunological tumour subtypes.

## Conclusions

There was an association between peri-tumoural spatial distribution of lipid composition with tumour cellular differentiation and proliferation. Peri-tumoural lipid composition imaging might have potential in non-invasive quantitative assessment of patients with breast cancer for treatment planning and monitoring.

## Supplementary Information

**Additional file 1.**

## Data Availability

The datasets generated and analysed during the current study are available from the corresponding author on reasonable request.

## Abbreviations

MRI: Magnetic resonance imaging
MUFA: Monounsaturated fatty acids
PUFA: Polyunsaturated fatty acids
SFA: Saturated fatty acids
