# Supplementary material for: Peri-tumoural spatial distribution of lipid composition and tubule formation in breast cancer
Source: BMC Cancer. 2022 Mar 17;22:285. doi: 10.1186/s12885-022-09362-1 (PMC8928628; doi:10.1186/s12885-022-09362-1)
Supplement: Supplementary file 1 — Additional file 1. [file 12885_2022_9362_MOESM1_ESM.pdf]

[Supplementary Materials:]

Data Acquisition: Data acquisition was performed on a 3T MRI scanner (Achieva TX, Philips Healthcare, Best, Netherlands) using 3D multi-echo gradient echo (GRE) sequence with 16 echoes, initial TE of 1.14 ms, echo spacing of 1.14 ms, TR of 20 ms, resolution of 2.2 mm isotropic, flip angle of 6° and 9 signal averages. The total acquisition time was 8.5 minutes.

Phantom Experiments: Quantitative maps of individual lipid constituent were obtained from six oil phantoms: cod liver oil (Sainsbury's, UK), corn oil (Mazola, USA), olive oil (Sainsbury's UK), peanut oil (Sainsbury's, UK), soy oil (Pride, UK) and sunflower oil (Flora, UK). Experiments for sunflower oil phantom were performed in triplicates for reproducibility. Identical data acquisition protocol was used *in vitro* and *ex vivo*.

Data Processing: Data were processed in MATLAB (MathWorks Inc., Natick, MA, USA) using in-house software. Chemical shift-encoded imaging (CSEI) considers the multi-echo GRE signal, S, at echo time (TE) as the sum of the water and lipid signal (S_{water} and S_{lipid}),

$$S(TE) = \left| S_{water} e^{i\omega_{water}TE} + S_{lipid} \sum_{n}^{N} \alpha_n e^{i\omega_n TE} \right| e^{-R_2^* TE} \qquad [Eq.S1]$$

where ω_{water} is the angular water frequency shift, $\sum_n^N \alpha_n e^{\omega_n TE}$ describes the multi-frequency lipid spectrum with angular frequency ω_n and relative amplitude α_n for lipid peak *n* [1], and the overall signal decays with a single transverse relaxation rate R_2* [2]. Conventional CSEI utilises a pre-calibrated lipid spectrum with fixed amplitudes for fat fraction mapping [3], while

spectroscopic approach estimates the individual lipid signal amplitude in composition analysis [4]. Lipid composition mapping by CSEI can be achieved through incorporating the spectral characteristics of the molecular structure of triglyceride to the relative amplitude within the model, described as carbon chain length (*CL*), number of double bonds (*ndb*) (-CH=CH-) and number of methylene-interrupted double bonds (*nmidb*) (-CH=CH-CH_2-CH=CH-) [4,5]. The descriptive characteristics of the model were further simplified empirically as derivatives of number of double bonds [6] as:

$$CL = 16.8 + 0.25 \times ndb \qquad [Eq. S2], and$$

$$nmidb = 0.093 \times ndb^2 \qquad [Eq. S3].$$

Hence, the maps of number of double bonds were computed using multi-peak spectrum model established in liver [4], thigh [5] and breast adipose tissue [7,8], with the relative frequency to water shifted by 0.2 ppm for lower experimental temperature (20°C) compared to *in vivo* condition [9], summarised in Table S1. Quantitative maps of individual lipid constituent as a percentage of the total amount of lipids were subsequently computed based on the maps of number of double bonds and Eqs. S2-S3 [5]:

$$PUFA = \frac{nmidb}{3} \qquad [Eq. S4],$$

$$MUFA = \frac{ndb - nmidb}{3} - PUFA \qquad [Eq. S5], and$$

$$SFA = 1 - PUFA - MUFA \qquad [Eq. S6].$$

In oil phantoms, the region of interest (5×5×5 voxel^3) was delineated at the centre of oil on the first echo of lipid composition images. The mean *ndb* and individual lipid constituent were calculated as the arithmetical mean of *ndb* and individual lipid constituent from the region of interest in the quantitative

maps. The individual lipid constituent computed from CSEI provided good approximation against values from manufacturer's nutrition label (Figure S1).

In breast tumours, the peri-tumoural region was delineated on the first echo of lipid composition images, and adipose voxels (lipid signal over 60 % of total signal) within the region were extracted from lipid composition maps for histogram analysis (Figure S2). The spatial distribution (mean, skewness, entropy and kurtosis [9,10]) were subsequently computed based on histogram distribution for each lipid constituent (Table S2). Lipid constituent mean was calculated as the arithmetical mean of lipid constituent from all the voxels in the peri-tumoural region. Skewness was calculated as the asymmetry of lipid constituent distribution from all the voxels on histogram [11]. Entropy was calculated as the irregularity of lipid constituent distribution on histogram [10,11]. Kurtosis was calculated as the peakedness of the histogram distribution [11]. The mathematical functions *mean*, *skewness*, *entropy* and *kurtosis* were deployed in MATLAB, with details in a review [12].

[Supplementary Table:]

Table S1. Multi-peak spectrum model.

The spectral peak chemical species, frequency (relative to water) and relative amplitude. The amplitudes of individual lipid peaks are expressed as a function of number of double bonds (*ndb*) in triglycerides. A frequency shift of 0.2 ppm between lipid and water is incorporated to account for lower temperature at 20°C [9].

Spectral peaks		Chemical species	Frequency (ppm) (ω_n)	Signal amplitude×100 (α_n)
**Water**		$\mathbf{H}_2O$	0.0	-
**Lipid**	Olefinic and Glycerol	-C**H**=C**H**- / -C**H**-O-CO-	0.4/0.3	2×*ndb*+1
	Glycerol	-C$\mathbf{H}_2$-O-CO-	-0.7	4
	Diacyl	-CH=CH-C$\mathbf{H}_2$-CH=CH-	-2.15	2×(0.093ndb^2)
	α-Carboxyl	-CO-C$\mathbf{H}_2$-CH_2-	-2.6	6
	α-Olefinic	-C$\mathbf{H}_2$-CH=CH-C$\mathbf{H}_2$-	-2.8	(*ndb*-0.093ndb^2)×4
	β-Carboxyl and Methylene	-CO-CH_2-C$\mathbf{H}_2$- / -(C$\mathbf{H}_2$)$_n$-	-3.3/-3.6	6+((16.8+0.25*ndb*)-4)×6 - (*ndb*×8)+ 0.093ndb^2×2
	Methyl	-$(CH_2)_n$-C$\mathbf{H}_3$	-4.0	9

ndb: number of double bonds

Table S2. A description of spatial distribution measures (heterogeneity) of lipid constituent in the peri-tumoural region [12].

Measures	Definition	Mathematical expression
Mean	Arithmetical mean of lipid constituent from all the voxels in the region of interest	$\bar{a} = \frac{1}{n}\sum_{(x,y)}[a(x,y)]$<br>*Image: *a(x,y)*
Skewness	1. Third standardised moment of the selected voxels<br>2. Reflects the asymmetry of lipid constituent distribution from all the voxels on histogram	$s = \frac{n}{(n-1)(n-2)}\frac{\sum_{(x,y)}[a(x,y) - \bar{a}]^3}{[sd(a)]^3}$
Entropy	1. Frequency of occurrence (probability) of all grey levels in the selected voxels<br>2. Reflects the irregularity of lipid constituent distribution from all the voxels on histogram	$e = -\sum_{l=1}^{k}[p(l)]log_2\,[p(l)]$<br>*Probability of the occurrence of the voxel value *(l)*: *p(l)*
Kurtosis	1. Fourth standardised moment of the selected voxels<br>2. Reflects the peakedness of lipid constituent distribution from all the voxels on histogram	$k = \frac{n(n+1)}{(n-1)(n-2)(n-3)}\frac{\sum_{(x,y)}[a(x,y) - \bar{a}]^4}{[sd(a)]^4} - 3\frac{(n-1)^2}{(n-2)(n-3)}$

[Supplementary Figures:]

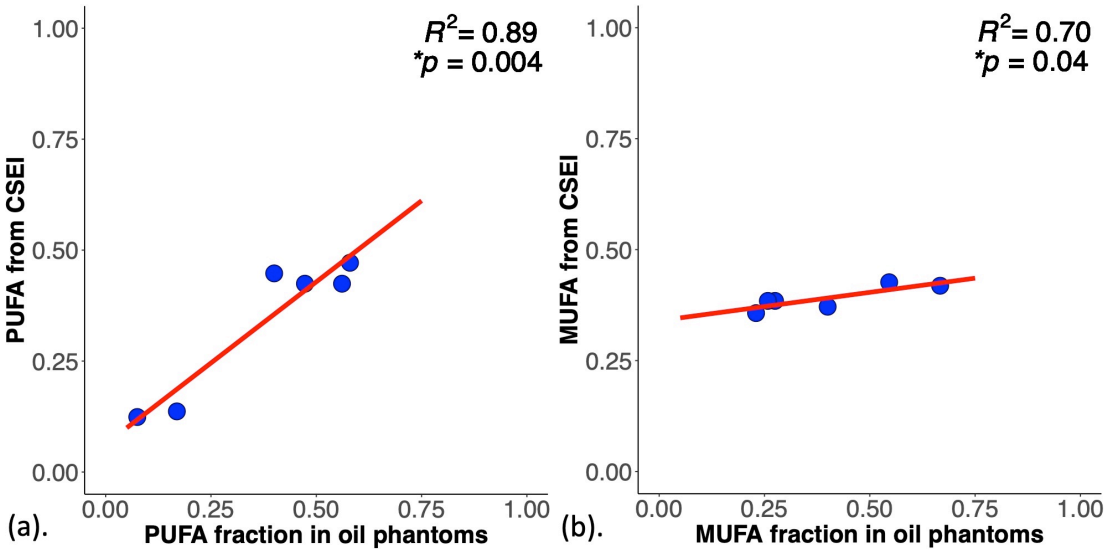

Figure S1. Correlation of (a) polyunsaturated fatty acids (PUFA) and (b) monounsaturated fatty acids (MUFA) from chemical shift-encoded imaging (CSEI) against manufacturer's fatty acid fractions in six oil phantoms.

The coefficients of determination (R^2) are above 0.70 for PUFA and MUFA, with $p < 0.05$, indicating significant linear correlation between the quantification of lipid composition from CSEI against fatty acid fractions in six oil phantoms (cod liver, corn, olive, peanut, soy, sunflower).

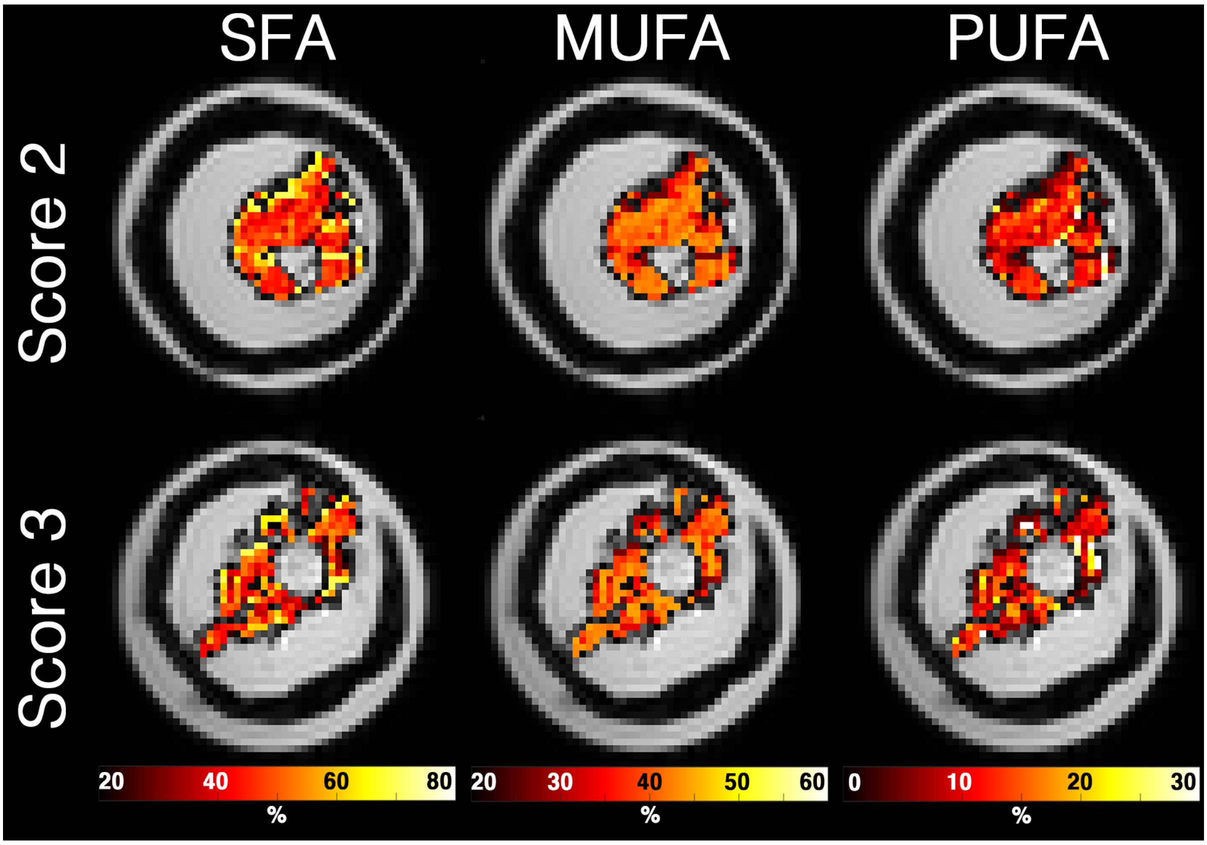

Figure S2. Peri-tumoural lipid composition maps from typical specimens of different tubule formation scores.

Freshly excised whole breast tumours were submerged in formalin and immobilised using a dedicated hold harness. The specimen was positioned at the isocentre of the scanner to acquire lipid composition images using chemical shift-encoded imaging. The saturated fatty acid map (left), monounsaturated fatty acid map (middle) and polyunsaturated fatty acid map (right) for tubule formation Score 2 and 3 are shown.

References:

1. Yu H, Shimakawa A, McKenzie CA, Brodsky E, Brittain JH, Reeder SB. Multiecho water-fat separation and simultaneous R_2* estimation with multifrequency fat spectrum modeling. Magn Reson Med. 2008;60(5):1122–1134.

2. Hernando D, Liang Z-P, Kellman P. Chemical shift-based water/fat separation: A comparison of signal models. Magn Reson Med. 2010;64(3):811–822.

3. Reeder SB, Robson PM, Yu H, Hernando, D, Pineda, AR. Quantification of hepatic steatosis with MRI: the effects of accurate fat spectral modeling. J Magn Reson Imag. 2009; 29(6):1332–1339.

4. Hamilton G, Yokoo T, Bydder M, Cruite, I, Schroeder, ME, Sirlin, CB, et al. In vivo characterization of the liver fat ^{1}H MR spectrum. NMR Biomed. 2011;24(7):784–790.

5. Peterson P, Månsson S. Simultaneous quantification of fat content and fatty acid composition using MR imaging. Magn Reson Med. 2013;69(3):688–697.

6. Bydder M, Girard O, Hamilton G. Mapping the double bonds in triglycerides. Magn Reson Imag. 2011;29(8):1041–1046.

7. Coum A, Ouldamer L, Noury F, Barantin, L, Saint-Hilaire, A, Vilde, A, et al. In vivo MR spectroscopy of human breast tissue: quantification of fatty acid composition at a clinical field strength (3 T). Magma. 2016;29(1):1-4.

8. Dimitrov IE, Douglas D, Ren J, Smith, NB, Webb AG, Sherry AD, et al. In vivo determination of human breast fat composition by ^{1}H magnetic resonance spectroscopy at 7 T. Magn Reson Med. 2012;67(1):20-26.

9. Bydder M, Hamilton G, de Rochefort L, Desai, A, Heba, ER, Loomba, R, et al. Sources of systematic error in proton density fat fraction (PDFF) quantification in the liver evaluated from magnitude images with different numbers of echoes. NMR Biomed. 2018;31(1):e3843.

10. Kim J-H, Ko ES, Lim Y, Lee KS, Han B-K, Ko EY, et al. Breast cancer heterogeneity: MR imaging texture analysis and survival outcomes. Radiology. 2017; 282(3): 665–675.

11. Just N. Improving tumour heterogeneity MRI assessment with histograms. Br J Cancer. 2014;111(12): 2205–2213.

12. Davnall F, Yip CSP, Ljungqvist G, Selmi M, Ng F, Sanghera B, et al. Assessment of tumour heterogeneity: an emerging imaging tool for clinical practice? Insights Imag. 2012;3(6): 573–589.
